# Loggerhead Turtles (*Caretta caretta*) Use Vision to Forage on Gelatinous Prey in Mid-Water

**DOI:** 10.1371/journal.pone.0066043

**Published:** 2013-06-12

**Authors:** Tomoko Narazaki, Katsufumi Sato, Kyler J. Abernathy, Greg J. Marshall, Nobuyuki Miyazaki

**Affiliations:** 1 International Coastal Research Center, Atmosphere and Ocean Research Institute, The University of Tokyo, Kashiwa, Chiba, Japan; 2 National Geographic Remote Imaging, Washington, D.C., United States of America; 3 Ocean Policy Research Foundation, Tokyo, Japan; University of St Andrews, United Kingdom

## Abstract

Identifying characteristics of foraging activity is fundamental to understanding an animals’ lifestyle and foraging ecology. Despite its importance, monitoring the foraging activities of marine animals is difficult because direct observation is rarely possible. In this study, we use an animal-borne imaging system and three-dimensional data logger simultaneously to observe the foraging behaviour of large juvenile and adult sized loggerhead turtles (*Caretta caretta*) in their natural environment. Video recordings showed that the turtles foraged on gelatinous prey while swimming in mid-water (i.e., defined as epipelagic water column deeper than 1 m in this study). By linking video and 3D data, we found that mid-water foraging events share the common feature of a marked deceleration phase associated with the capture and handling of the sluggish prey. Analysis of high-resolution 3D movements during mid-water foraging events, including presumptive events extracted from 3D data using deceleration in swim speed as a proxy for foraging (detection rate = 0.67), showed that turtles swam straight toward prey in 171 events (i.e., turning point absent) but made a single turn toward the prey an average of 5.7±6.0 m before reaching the prey in 229 events (i.e., turning point present). Foraging events with a turning point tended to occur during the daytime, suggesting that turtles primarily used visual cues to locate prey. In addition, an incident of a turtle encountering a plastic bag while swimming in mid-water was recorded. The fact that the turtle’s movements while approaching the plastic bag were analogous to those of a true foraging event, having a turning point and deceleration phase, also support the use of vision in mid-water foraging. Our study shows that integrated video and high-resolution 3D data analysis provides unique opportunities to understand foraging behaviours in the context of the sensory ecology involved in prey location.

## Introduction

Foraging is a series of processes by which animals acquire energy and nutrients. Foraging can be divided into functional units, including the search for, assessment, pursuit, and handling of the prey [1]. Foraging plays a central role in the ecology of animals affecting their survival, growth, and reproductive success [2]. Loggerhead turtles, *Caretta caretta* (Linnaeus, 1758), are long-lived marine reptiles that are widely distributed in temperate to tropical waters. The foraging ecology of loggerhead turtles has traditionally been studied by means of the analysis of contents of the digestive tract and faeces, providing lists of prey items consumed by turtles at various life stages (e.g., [3], [4]). Loggerhead turtles exhibit ontogenetic shifts in diet and habitat [5]. In the early life stage, juveniles remain in the epipelagic zone, foraging primarily on gelatinous zooplankton and other small invertebrates [4]. After spending some years in this oceanic habitat, large juveniles recruit to neritic habitats and shift to a diet composed primarily of hard-shelled benthic invertebrates [4], [6], [7], such as molluscs, crabs and barnacles; this shift corresponds with an increase in bite performance during this developmental period [8]. However, recent studies using satellite telemetry and stable isotope analysis have revealed that this ontogenetic shift in diet and habitat is facultative [9]–[11] and could be reversible [12], suggesting that the foraging ecology of loggerhead turtles is more complex than once believed. A number of studies have discussed the foraging ecology of loggerhead turtles in terms of what and where they forage. However, there is a paucity of information about how these animals search out, capture, and handle their prey. To further understand the life history of these turtles, it is important to examine their actual foraging behaviour.

However, direct observation of foraging behaviour among marine animals, including sea turtles, is logistically difficult, and only a few reports are available of activities in shallow water [13], [14]. Nonetheless, with recent development in microelectronic technology, several new methods have been explored. For example, Wilson et al. [15] introduced an inter-mandibular angle sensor using a Hall sensor-magnet system attached to each side of the mandible (IMASEN). Using the inter-mandibular angles as indicators of prey ingestion, this technology has been used to examine the underwater foraging activities of various marine animals, such as penguins [16]–[18], pinnipeds [19], and sea turtles [20], [21]. Alternatively, dynamic movements associated with attempts at capturing prey can be monitored using miniaturized accelerometers attached to the mandible [22]–[24] or the head [25]. Although these methods provide novel insights into the frequency and timing of foraging events, how the animals forage and on what remains unknown.

The use of animal-borne imaging systems has proven useful for studying various underwater behaviours [26]–[28], including the foraging behaviour of pinnipeds [29]–[31], penguins [32], [33], and sea turtles [34]. By recording foraging activities from the predator’s point of view, this technology can provide new insights into not only the predator’s prey (e.g., [26], [35]) but also its foraging habitat [32], [36] and prey density [37]. However, the imperfect lighting of these imaging systems and limited memory sizes often restrict their use to relatively short durations in sufficiently lit environments.

Conversely, 3D data loggers can be used regardless of the intensity of ambient light. The data obtained from 3D data loggers enable researchers to reconstruct 3D underwater movements by dead-reckoning using locomotion vectors: heading, depth (or pitch angle), and swim speed. As aquatic animals forage in a 3D environment, this method is useful for describing fine-scale movements during a foraging event, such as turns and bursts of speed while approaching prey [29], [38]. By examining predators’ track, it is also possible to provide insights of sensory cues involved in prey detection [39]. For example, tracks of predators primarily depending on visual search can occur in straight lines whereas that of odour-guided searchers tend to be zigzag shape because they make a number of turns to move within a odour plume to locate the source [40]. In the present study, we used an animal-borne imaging system and 3D data logger simultaneously to identify characteristics of behaviours during foraging events. Based on these characteristics, foraging events were extracted from 3D data when video data were unavailable. By examining in detail the series of movements made during these foraging events, we aim to discuss how loggerhead turtles forage in natural environments.

## Materials and Methods

### Ethics Statement

This study was conducted as a part of tag and release program in which loggerhead turtles caught by set net as bycatch in Iwate Prefecture, Japan, were turned over by fishermen to researchers. This study was performed in accordance with the guidelines of the Animal Ethic Committee of the University of Tokyo, and the protocol of the study was approved by this committee (Permit No. P05-5). Instruments were attached to the carapaces of the turtles using automatic time-scheduled releasers (Little Leonardo Co., Tokyo, Japan).

### Study Site and Animals

Fieldwork was conducted in the coastal waters of Sanriku on the northern Pacific coast of Japan, a seasonal foraging ground for loggerhead turtles to which turtles migrate during summer and autumn [41] despite the absence of a proximate nesting ground. During a period from 2006 to 2009, we collected turtles from fishermen when the turtles were incidentally captured in local set nets distributed between Miyako and Ofunato (38°55′−39°40′N, 141°40′−142°05′E). All turtles were promptly transferred to tanks at the International Coastal Research Center, the University of Tokyo (39°21′05N, 141°56′04E), where they were retained from 1 week to up to 3 months to collect faecal samples. While in captivity, turtles were fed on soft tissue of squid. The turtles ranged from 553 to 850 mm (mean ± s.d. = 737±91 mm, *N = *12) in standard carapace length (SCL) and from 27.9 kg to 94.5 kg (mean ± s.d. = 61.6±18.8 kg) in body mass (Table 1). Given that the SCL of adult females nesting on the Japanese coast was ≥692 mm [42], the turtles captured from the study site were assessed as juvenile to adult-sized turtles. Sex was determined as male only when an obvious extension of the tail (tail length >300 mm) was observed in large turtles (SCL>700 m). Otherwise, sex was not determined. After the deployment of multisensor data loggers, all turtles were released from Otsuchi Bay, Iwate, Japan (39°20N, 141°56E) using the research boat *Challenger III* of the International Coastal Research Center.

**Table 1:** Summary of deployments.

Turtle ID	SCL (mm)	Body mass (kg)	Sex	Logger type**	3D data (h)	Video data (h)	No. of foraging event recorded by video
L0601	728	61.6	Male	3D	6.2	–	–
L0602	788	65.5	Male	3D	14.4	–	–
L0603	588	32.8	Unknown	3D	14.3	–	–
L0609	836	76.0	Male	3D	15.6	–	–
L0704	700	60.5	Unknown	3D+C_1_	16.8	4.5	1
L0705	800	83.0	Unknown	3D+C_1_	15.8	2.0	1
L0708	730	54.5	Unknown	3D+C_1_	16.4	4.5	20
L0711*	850	94.5	Unknown	3D+C_1_	13.5	–	–
L0711*	850	94.5	Unknown	3D+C_1_	4.9	4.9	0
L0801	778	63.0	Unknown	3D+C_2_	32.2	2.0	3
L0825	707	53.0	Unknown	3D	21.4	–	–
L0832	553	27.9	Unknown	3D	22.3	–	–
L0947	781	67.0	Male	3D+C_2_	17.0	3.6	50

* L0711 was used for the study twice because it was recaptured by a set net after the first deployment.

** Abbreviations were used for logger type: 3D (W1000-3MPD3GT), C_1_ (Crittercam Gen 5.5) and C_2_ (Crittercam Gen. 5.7).

### Data Loggers and Fieldwork Procedure

To record fine-scale underwater movements, we attached a 3D logger (W1000-3MPD3GT; 26 mm in diameter, 175 mm in length, 140 g in air; Little Leonardo Co., Tokyo, Japan) to the carapace of each turtle. The 3D loggers were programmed to record depth, temperature, swim speed, and tri-axis magnetisms at 1 Hz, and tri-axis accelerations at 32 Hz. In addition to the 3D loggers, animal-borne imaging systems ‘Crittercam’ (76 mm in diameter, 350 mm in length, 1.5 kg in air for Gen. 5.5; and 57 mm in diameter, 230 mm in length, 0.8 kg in air for Gen. 5.7; National Geographic – Remote Imaging, Washington DC, USA) were used in seven deployments (Table 1). The Crittercam consists of a microprocessor-controlled video recorder (8-h maximum recording time), batteries, lights, VHF transmitter, microphone and pressure transducer contained in a waterproof and pressure-proof housing. We programmed the Crittercam to record video during the daytime when there was sufficient ambient light. The 3D logger, in contrast, recorded throughout deployment regardless of the time of day.

Both loggers needed to be retrieved to obtain the data. As it was difficult to recapture the turtles, automatic time-scheduled release systems were used (see details in [43]). Once the release system was activated, the loggers were located via VHF radio signal using a Yagi antenna and a receiver, and were recovered by the R/V *Yayoi* of the International Coastal Research Center.

### 3D Path Reconstruction and Visual Data Analysis

Time-series data obtained from the 3D loggers were analysed using IGOR Pro ver. 6.04 (WaveMatrics, Lake Oswego, OR, USA). In this study, a dive was defined as any submergence to a depth of >1 m. Based on the shapes appearing in the time-series depth plot, dives were classified into four types corresponding to Houghton et al. [44] and Seminoff et al. [34]: U-shaped dives (Type 1), V-shaped dives (Type 2), gradual ascent dives without a steep initial ascent phase (Type 3) and gradual ascent dives with a steep initial ascent phase (Type 4). Any subsurface dives shallower than 4 m were excluded from the analysis. 3D paths were calculated using data on swim speed, acceleration, and magnetism obtained from the 3D loggers, as described in Narazaki et al. [43]. The speed sensor of the 3D logger recorded swim speed as the rotation of an external impeller mounted on the anterior end of the logger, which strongly correlated with the speed of water flow passing through the impeller. The number of rotation per second of the impeller was converted into speed (m s^−1^) using a regression line obtained for each logger (no. of points in the regression ≥5 and *R* ^2^≥0.95 for all loggers). In addition, swim speeds lower than the stall speed of 0.2 m s^−1^ were considered indistinguishable from zero in this study. The acceleration sensors of the 3D loggers recorded both specific accelerations (e.g. flipper movements) and gravity-based accelerations (i.e., changes in response to posture change). Under the assumption that changes in posture occur at a lower frequency than changes in accelerations resulting from body motions such as thrust, frequency-based filters (0.19–0.28 Hz low-pass finite impulse response filters) were applied to the entire acceleration dataset to separate it into two components. Then, the pitch and roll angles of the turtles were calculated from the low-frequency components of accelerations. The high-frequency component of longitudinal acceleration was used to analyse flipper movements. Given that the magnetism values recorded by the loggers varied depending on the angles between the geomagnetic vector and each axis of the loggers, headings were calculated from pitch, roll and tri-axis magnetism [45] using the macro ThreeD_path [46] (available at: http://bre.soc.i.kyoto-u.ac.jp/bls/index.php?3D_path), which was compatible with IGOR Pro (WaveMatrics). Thereafter, 3D paths were reconstructed using the dead-reckoning method [47]–[49].

The Crittercams recorded video data in MPEG4-DivX video format. Video data were thoroughly inspected by using a DivX player (DivX, San Diego, CA, USA) to determine any foraging events. A foraging event was extracted when the turtle bit on a single prey or a series of prey (e.g., a chain of siphonophores). Then, underwater behaviours during foraging events were analysed by linking the video data with 3D paths.

### Extraction of Turning Point

**Figure 1:**
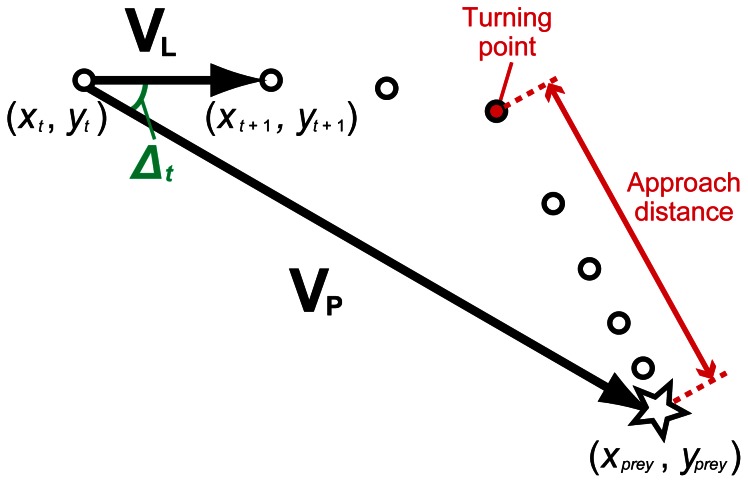
A schematic diagram of horizontal movements during a foraging event. Open dots represent the location of a turtle obtained from the 3D logger every second. The location where the turtle captured the prey is represented as a star. The delta angle (Δ*_t_*) between the locomotion vector at time *t* (**V_L_**) and the prey vector (**V_P_**) was calculated every second to determine the turning point (red dot).

By examining horizontal paths during foraging events, we observed that turtles changed their direction of travel toward prey in some events. To determine the turning point at which the turtle turned toward the prey, we calculated for each second the difference in angle between the travel direction of the turtle and the direction toward the prey (hereafter, the “delta angle”; Fig. 1). When the location of the turtle at a given time *t* and that of the prey in the horizontal plane were (*x_t_, y_t_*) and (*x_prey_, y_prey_*), respectively, the locomotion vector at *t* (**V_L_**) and the vector from the location of a turtle at *t* and the prey (**V_P_**) can be expressed as follows (see Fig. 1):
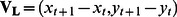
(1)

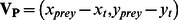
(2)


Then, the delta angle at given time t (Δ*_t_*) can be calculated as
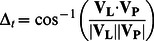
(3)


For each foraging event, Δ*_t_* was computed for each second backward from the bite point to the point 60 s prior to the bite point. The turning point was determined as the first point at which Δ*_t_* exceeded 15°. The period between the turning point and the bite point was defined as approach phase. When all Δ*_t_* were ≤15°, it was considered that there was no turning point during the event (i.e., no approach phase). For the events with approach phases, the distance between the turning point and the bite point (hereafter, “approach distance”; Fig. 1) was calculated.

### Extraction of Potential Foraging Events from 3D Data

**Figure 2:**
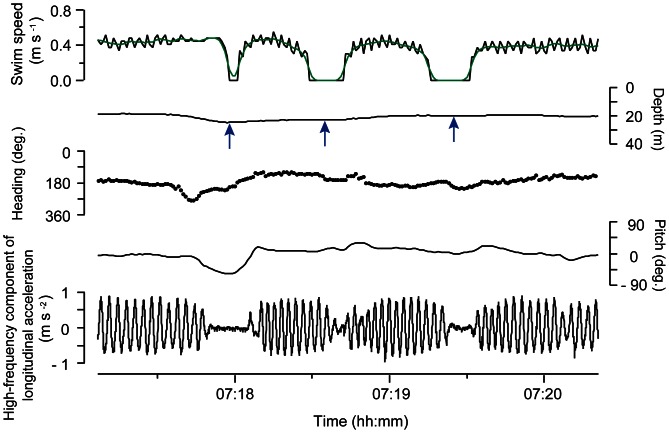
Time-series behavioural data showing conspicuous drops in swim speed during mid-water foraging events. Blue arrows indicate the time at which the animal-borne video recorded the prey capture (bite point). The green line is smoothed speed data used for the extraction of presumptive events. Periodic movements shown in high-frequency component of longitudinal acceleration reflect flipper movements (i.e., strokes).

In this study, video data recorded a limited amount of daytime activity, whereas 3D data could record for a longer duration, regardless of whether it was day or night. By examining the 3D movements during foraging events recorded by video data (hereafter, “true event”), we found that foraging events shared a common feature of turtles slowing down to capture their prey (Fig. 2). Based on this conspicuous deceleration associated with foraging, potential foraging events were extracted from the 3D data as the periods when the swim speed dropped below a certain threshold (hereafter, “presumptive events”). To determine the threshold, we first extracted presumptive events from 3D data when video data were also available. Time-series swim speed data showed zigzag shapes, of which peaks reflected accelerations by strokes (see Fig. 2). Thus, swim speed data were smoothed by 4 s to remove the effect of instantaneous drops in swim speed, and the mean and s.d. of the smoothed speed during dives were obtained for each deployment. In this study, four thresholds were tested: mean speed minus s.d. multiplied by 1, 1.5, 2, and 2.5. The duration of presumptive events to be extracted was restricted to 5–90 s based on the observations of the video data. To select the best threshold, we calculated the detection rate (the number of true events successfully extracted divided by the total number of true events) and the false detection rate (the number of presumptive events incorrectly extracted divided by the total number of presumptive events) for each threshold. Then, the best threshold which produced the largest sum of detection rate and 1– false detection rate, was selected [33]. Finally, presumptive events were extracted from 3D data when video data was absent. The bite point for each presumptive event was defined as the first point at which swim speed reached the minimum value. Turning points during presumptive events were extracted in the same manner as those from true events.

### Statistical Analysis

Combining presumptive foraging events and the true events occurring in mid-water (>1 m), we examined the characteristics of mid-water foraging events. Generalised linear mixed models (GLMM) in the R package (The R project for Statistical Computing) were used to compare the characteristics of mid-water foraging events between true and presumptive events and between daytime and night-time events. Mixed models were used because data obtained from the same turtle were not independent. Hence, turtle ID was treated as a random variable. A model was selected for each of the dependent variables: foraging depth, approach distance and the presence of a turning point. Explanatory variables for the depth model were i) the nature of the event (i.e., true or presumptive) and ii) the time of day (i.e., day or night). In addition to these two variables, iii) the depth at the bite point was used as an explanatory variable for the other models. For the depth and the approach distance models, the gamma error and log-link function were used. The binomial error and the logit link function were used for the presence of the turning point model. The most parsimonious model was selected on the basis of Akaike Information Criteria (AIC) for each model, and chi-square analysis of deviance was used to determine the effect of terms in the selected model.

## Results

Animal-borne video cameras were used in seven deployments. However, we had trouble recording video data in one deployment (L0711). As a result, a total of 21.5 h of video data were obtained from six deployments. A total of 210.8 h of 3D data were obtained from 12 turtles over 13 deployments. A total of 816 dives were recorded, which were classified into four types: Type 1 (depth = 20 m and 34.8 m, duration = 1964 s and 5144 s, respectively, *N* = 2), Type 2 (mean depth ± s.d. = 18.5±17.6 m, mean duration ± s.d. = 215±227 s, *N* = 402), Type 3 (18.4±10.2 m, 1107±791 s, *N* = 260) and Type 4 (34.9±27.6 m, 969±806 s, *N* = 152).

### Foraging Behaviour Observed from Video and 3D Data

**Figure 3:**
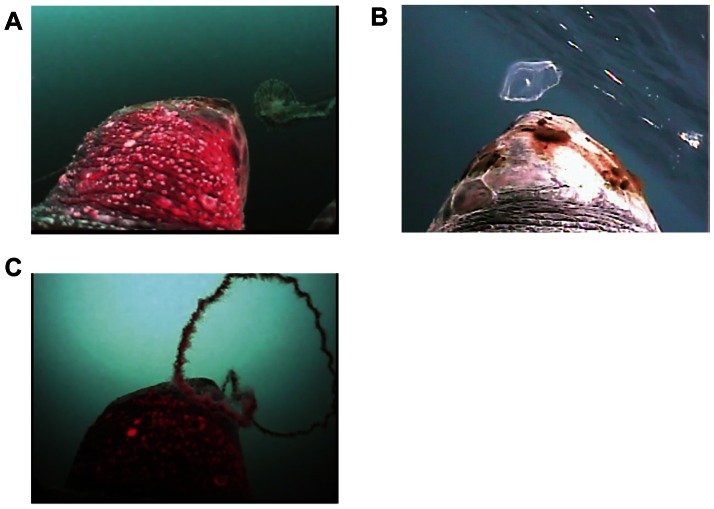
Examples of gelatinous prey. **A**. *Chrysaora melamaster*, **B**. *Aequorea coerulescens*, **C**. A chain of siphonophores.

A total of 75 foraging events involving five turtles were video recorded. Foraging events were recorded at a mean depth of 19.7±9.6 m. Most of events were recorded while the turtles were actively swimming either in mid-water (i.e., epipelagic water column deeper than 1 m) or at the sea surface (≤1 m). Seventy-one out of the 75 events were associated with gelatinous organisms, such as *Chrysaora melanaster* (8 events), *Aequorea coerulescens* (1 event), and some siphonophores (62 events) that could not be identified to the species level (Fig. 3, also see Movie S1, S2). In one foraging event, the turtle (L0704) foraged on a lump of seaweed at the sea surface that was incidentally detached from its own forelimb. Only one turtle (L0947) performed three foraging events at the sea bottom (mean depth ± s.d. = 44.9±0.1 m, *N* = 3). However, the prey items could not be identified because of insufficient light.

By linking the video and 3D data, we examined mid-water foraging behaviours. We excluded surface events (≤1 m; 11 events) from this analysis because swim data were not reliable at the sea surface, as once the impeller of the 3D logger came out of the water, it did not rotate as well as it did in the water. In addition, no reliable swim data were obtained for 30 out of 61 mid-water events because the impeller of the 3D logger got entangled with food particles. As a consequence, 31 out of 61 mid-water foraging events were used for the analysis. Excluding the periods for which swim speed data were unreliable, the mean swim speed of the turtles during each deployment was 0.5±0.1 m s^−1^ (*N* = 6). In 26 out of the 31 events analysed, the turtles decelerated to below the threshold (i.e., mean speed – s.d.×2; determination of the threshold is discussed later) before they reached the prey and while handling the prey. We defined the period of deceleration before the prey was reached as the deceleration phase. The handling phase was defined as the period from the bite point to the point when the turtle’s speed recovered to the threshold. Characteristics of the deceleration and handling phases are summarized in Table 2.

**Table 2:** Characteristics of mid-water foraging events recorded by video data.

	No. of events analyzed	Depth (m)	Duration (s)	Swim speed* (m s^−1^)	Pitch (deg.)	Distance to prey (m)
Approach phase	23	22.2±4.2	22.5±13.9	0.3±0.1	−1.8±20.6	7.4±5.9
Deceleration phase	26	21.6±3.6	6.8±3.3	0.09±0.06	7.6±19.7	0.2±0.2
Handling phase	26	21.7±3.5	18.5±15.7	0.06±0.07	13.3±22.9	–

* Mean swim speed of each phase was calculated from speed data without applying any smoothing procedures.

**Figure 4:**
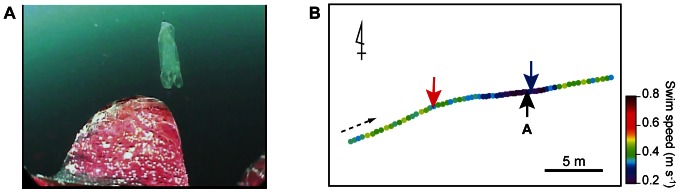
An encounter with a plastic bag. **A**. A plastic bag encountered by turtle L0708 while swimming in mid-water at a depth of 24.3 m. **B**. Horizontal movements made by the turtle while approaching the plastic bag. The dashed arrow shows the direction of the movement. The position where the turtle reached the plastic bag and the turning point are indicated by blue and red arrows, respectively. The black arrow indicates where the plastic bag appeared in the clip (Fig. 4A) which was 0.4 m before the turtle reached the plastic bag.

In addition, one individual, L0708, approached a plastic bag during our study (Movie S3). Although the turtle did not bite the plastic bag, it showed similar 3D movements to those of true foraging events associated with gelatinous prey (Fig. 4). Following the same definition as for the foraging events, the turning point and the start of the deceleration phase occurred at 9.6 m and 3.4 m, respectively, before the plastic bag was reached.

### Extraction of Presumed Foraging Events from 3D Data

**Figure 5:**
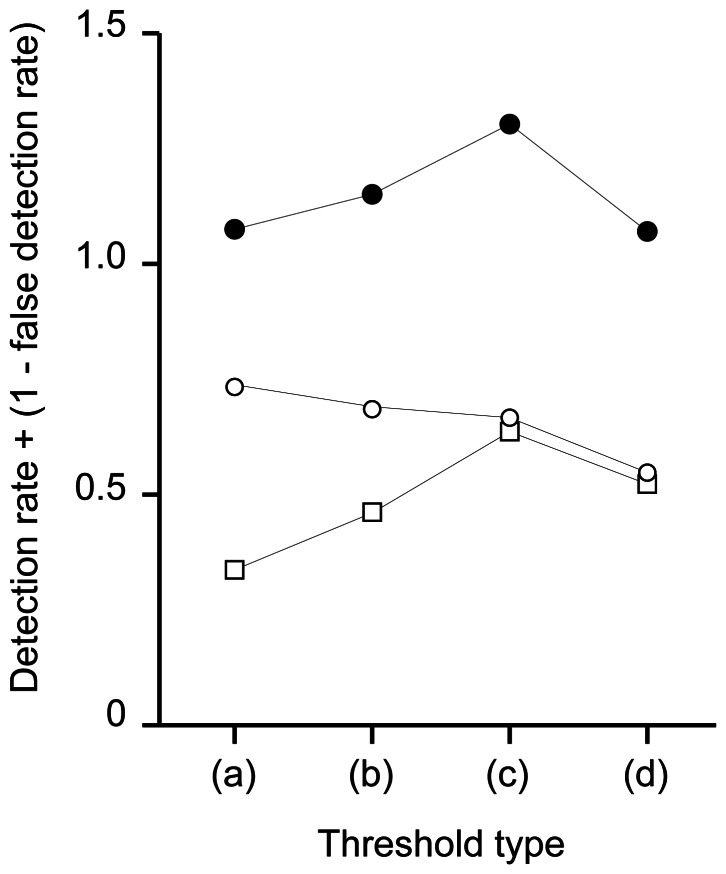
Comparison of five threshold types. The sums of detection rate and 1-false detection rate (closed circle), detection rate (open circle), and 1-false detection rate (open square) of four threshold types were compared: (a) mean speed – s.d., (b) mean speed – s.d.×1.5, (c) mean speed – s.d.×2, (d) mean speed – s.d.×2.5.

Based on the assumption that the turtles decelerated during mid-water foraging events, presumptive foraging events were extracted from the 3D data. The animal-borne video cameras recorded that the turtles sometimes approaching their potential prey without attempting to bite. As it seems that turtles made final decision not to feed after closely examining their potential prey, we treated these events as quasi-foraging events in this analysis (i.e., positive detection by 3D data was not treated as false). Among the four thresholds tested, the best threshold with the largest sum of detection rate and “1 – false detection rate” was selected as the mean swim speed of each deployment – s.d.×2 (Fig. 5), of which the detection and false detection rates were 0.67 and 0.36, respectively. For further analysis, using the selected threshold, we extracted a total of 369 presumptive events (102 daytime and 267 night-time events) from 169 h of 3D data for which video data were absent. For periods when both video and 3D data were recorded, 31 true mid-water foraging events were used.

### Foraging Behaviour during Daytime and Night-time

**Figure 6:**
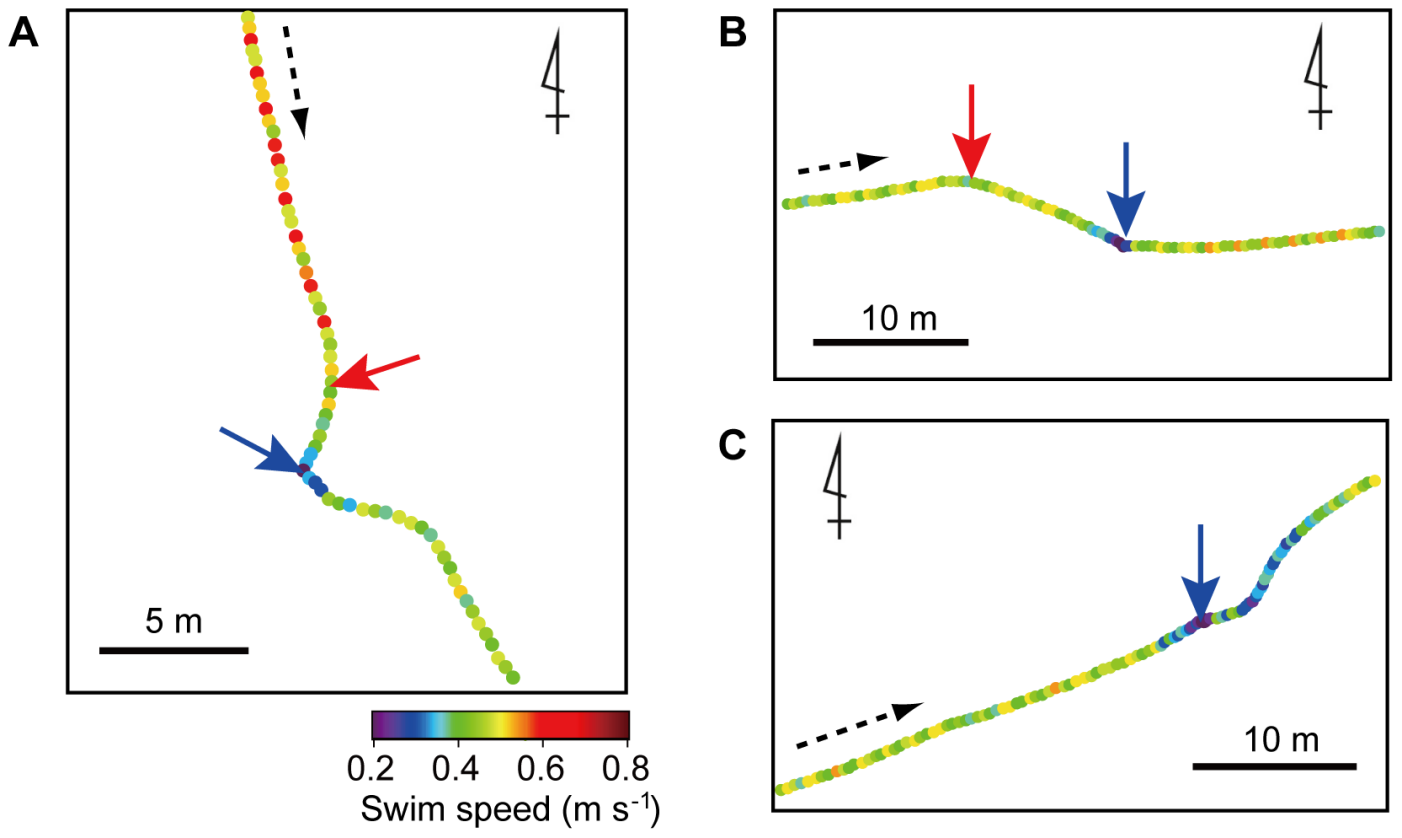
Horizontal movements during mid-water foraging events. Examples of foraging events recorded at 05∶04 on 20 August 2007 at a depth of 21.3 m (**A**), at 07∶15 on 3 September 2009 at a depth of 18.3 m (**B**), and at 05∶21 on 20 August 2007 at a depth of 18.3 m (**C**). A dashed arrow indicates the direction of movement for each path. Red and blue arrows indicate the turning point and the bite point. Note that there was no turning point in C.

**Figure 7:**
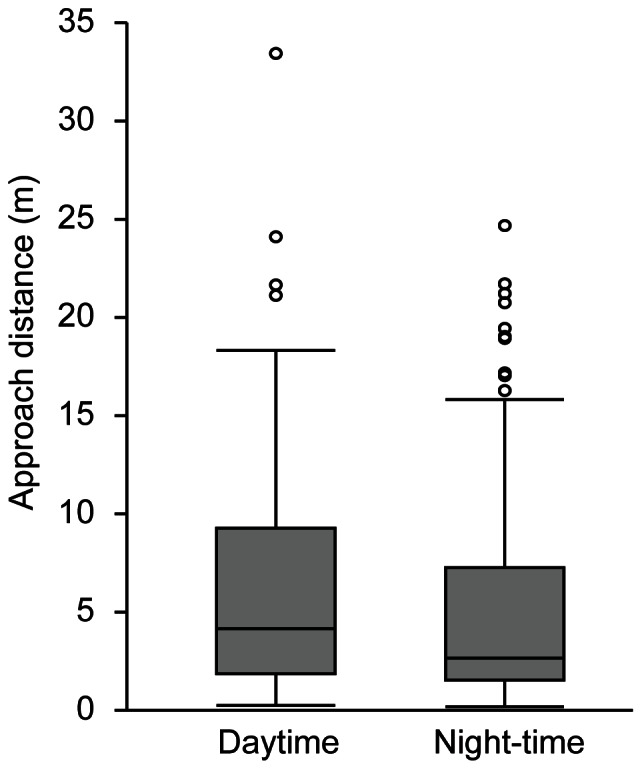
Comparison of approach distance during daytime and night-time events.

Combining the true and presumptive events, we analysed a total of 400 events. Most events were recorded during Type 3 dives (199 events) and Type 4 dives (166 events). Only 35 events occurred during Type 2 dives and no events occurred during Type 1 dives. The rate of events in mid-water (>1 m) was estimated as 2.1 times h^−1^ on average. However, events tended to occur not regularly but intensively. For example, a total of 53 mid-water events were recorded during 17 h of data for individual L0947. Forty-five of these events were recorded during a 3-h period (15 times h^−1^), and only eight events were recorded during the rest of the recorded period (0.6 times h^−1^). Mid-water events were recorded at a mean depth of 23.1±18.3 m (*N* = 400). In GLMM analysis, the most parsimonious model selected for event depth was one in which the variations were distributed randomly and were not related to either day/night or true/presumptive events (AIC = 163.5, Table S1). Turtles changed their direction toward prey an average of 5.7±6.0 m before reaching the prey in 229 events (Fig. 6A, B), whereas no such turning point was confirmed in 171 events (Fig. 6C). The presence of a turning point varied between day and night (GLMM, *χ^2^* = 5.16, *P* = 0.023). The probabilities of a turning point existing were estimated as 0.70 for daytime events and 0.58 for night-time events. For events with a turning point, the distance between the turning point and the prey (i.e., the approach distance) was related to both the event depth and day/night events (GLMM, AIC = 253.4, *χ^2^* = 7.68, *P* = 0.022, Table S1) but not to true/presumptive nature of the event. The GLMM analysis showed that approach distance was positively related to event depth (approach distance = e^0.009*depth +1.7^). Approach distance was larger in daytime events (median = 4.2 m, *N* = 86) than in night-time events (median = 2.6 m, *N* ***= ***143; Fig. 7).

## Discussion

Recent studies have shown that animal-borne imaging systems are useful tools for documenting various behaviours of free-living aquatic animals [26], [28], [35], [50]. Although it allowed for direct observation of foraging events, providing important information about prey and foraging habitats [34], [36], [51], there was a limitation in that video or still image data could only be recorded for a relatively short duration (up to 8 h) when sufficient ambient light was available. In contrast, 3D data could be obtained for a relatively longer duration regardless of the amount of light. Linking video to behavioural records (e.g., accelerations) can extend the effective duration of detecting prey capturing events [33]. To examine foraging events from entire data sets, video data was linked to 3D data to reveal a common behavioural signal associated with foraging. During dives, the turtles continuously stroked, maintaining a narrow range of swim speed (i.e., a cruise speed of 0.5 m s^−1^) that minimized the cost of transport [52]. However, when capturing their prey, the turtles interrupted their stroking and decelerated shortly before they reached the prey (Fig. 2). It has been suggested that aquatic predators change their pursuit speed depending on the type of prey to optimize foraging efficiency [38], [53], [54]. A decrease in speed during mid-water foraging by turtles could therefore be interpreted as a behavioural adaptation to capturing sluggish, gelatinous prey. Based on the presumption that periods of deceleration during dives were related to mid-water foraging activities, presumptive foraging events were extracted (detection rate = 0.67). The characteristics of foraging events in terms of foraging depth, the presence of a turning point, and approach distance did not differ between true and presumptive events (Table S1), which implies that decelerations can be used as effective proxies for mid-water foraging events. Foraging events in free-ranging sea turtles have been monitored by means of mouth-opening detected by magnetism [20], [21], or acceleration [23], which requires the attachment of more than one sensor near the mandible. Swim speed, in contrast, can be recorded by sensors on the carapace. The use of deceleration as an indicator of mid-water foraging events is a simple method that causes less disturbance to the animal. This might be applicable to turtles undertaking oceanic migrations which could allow mapping of high foraging success area [55], although development of data compression technique for relaying foraging signals via Argos satellite system is essential for long-term monitoring [56].

### Significance of Mid-water Foraging in Loggerhead Turtles

Our study shows that adult and large juvenile loggerhead turtles captured in a neritic environment perform numerous mid-water foraging events potentially associated with slow-moving gelatinous prey. It is important to note that our results do not controvert the importance of benthic prey in the turtles’ diet. A recent stable isotope analysis of juvenile loggerhead turtles in the Atlantic reported that oceanic jellyfishes contributed to their diet to some extent in both neritic and oceanic habitats [57]. In addition, faecal analysis conducted concurrently with our study found many benthic prey items, such as conches and sea urchins, especially in individual L0947 (Takuma & Narazaki, unpublished data), whose frequent mid-water foraging events were recorded in our study (Table 1). Despite the low energy density of jellyfish [58], the frequency of mid-water foraging observed in our study (2.1 times h^−1^ on average) suggests that traditional analysis of faecal and stomach contents have underestimated the importance of easily digestible gelatinous prey in the diets of loggerhead turtles.

Most mid-water foraging events were recorded during Type 3 and Type 4 dives, which shared a common feature of a prolonged gradual ascent phase. These dives could be considered travelling dives, as the turtles stroked continuously [43], [59] and remained at depth, attaining close to neutral buoyancy during the gradual ascent phase [60], [61]. Gradual ascent dives have been widely reported in marine turtles (e.g., [44], [60], [62], [63]), including during oceanic migrations [64]. In the open ocean, the distribution of gelatinous prey is highly patchy, but dense aggregations could be occasionally formed (e.g., [65]). Given that ectothermic animals, including sea turtles, have relatively low metabolic rate, energetic calculations suggested that they could obtain sufficient energy when they are in dense patches of gelatinous prey [66]. Thus, opportunistic foraging on gelatinous prey seems to be a good strategy for omnivorous loggerhead turtles especially during oceanic migrations when benthic preys are inaccessible to turtles at depths of thousands of metres.

### Potential Cues Used in Mid-water Foraging

Aquatic vertebrates use several types of cues, including chemical [67], [68], visual [69], [70], and auditory cues [71], [72] to find prey. Under laboratory conditions, the ability of sea turtles to detect and respond to the food odours has been demonstrated both underwater [73] and in the air [74]. The chemical substances emitted from prey diffuse across media (e.g., water), forming a gradient of different concentrations of chemicals. Thus, animals performing olfactory searches will repeatedly turn to move toward the higher concentrations of the odour plume (reviewed in [40]). Hence, the track of olfactory searches rarely follows a straight line but rather makes a zigzag shape [39], [40]. In the present study, turtles changed their travel direction toward prey an average of 7.4±5.9 m before reaching the prey (Table 2). However, after the turning point, turtles maintained a straight-line course to approach the prey (Fig. 6), suggesting that chemical cues may not have been the primary cues used. According to our results, the primary cues used in prey finding in mid-water foraging appear to be visual. Anatomical and electrophysiological studies have suggested that sea turtles have well-developed visual systems that provide better visual acuity in water than in air [75]. Under laboratory conditions, the underwater visual acuity of juvenile loggerhead turtles was estimated at 5.38 and 12.89 min of arc by using electrophysiological [76] and behavioural [77] approaches, respectively, suggesting that loggerhead turtles are capable of visually discerning prey underwater [76], [77]. If turtles use visual cues to detect prey, movements during mid-water foraging events should change depending on the intensity of the ambient light. In the aquatic environment, the intensity of light decreases exponentially with increasing depth. In the present study, however, the differences in behaviours arising from different foraging depths were presumably small, because most foraging events were recorded in a narrow range of depth (i.e., >80% of events occurred in water shallower than 30 m; see Fig. S1). However, movements during foraging events differed between daytime and night-time. During daytime events, a turning point was present 70% of the time, whereas night-time events were less likely to have a turning point. In addition, approach distances were longer during the daytime (Fig. 7), implying that turtles can detect prey from a greater distance during daytime. As foraging occurred at a mean depth of 23.2±18.3 m (*N* = 400) where sufficient ambient light was available during the daytime, it is suggested that turtles can visually detect prey from a distance and make a turn to approach the prey. At night-time, in contrast, turtles might not have been able to visually detect prey until they were very close to them. However, turning points were confirmed in some night-time events. Although there have been no reports of continuous bioluminescence in the gelatinous prey identified in our study (e.g., *Chrysaora melanaster, Aequorea coerulescens*), many species of ctenophores and siphonophores are bioluminescent, emitting light at wavelengths between 440 and 506 nm [78]. Because adult loggerhead turtles are responsive to wavelengths from 440 to 700 nm [79], the turtles might be able to detect the bioluminescent prey in the dark.

Our results show that the turtles decelerated shortly before attempting to capture gelatinous prey (Fig. 2), which might be a behavioural adaptation to capturing sluggish prey. During the deceleration phase, it is also possible that the turtles closely examine the potential prey to determine whether to capture the prey. In this study, we observed a turtle encountering a plastic bag (Movie S3). The movements of the turtle while approaching the plastic bag were analogous to those of a true mid-water foraging event, having a turning point and a deceleration phase (Fig. 4B). As the plastic bag resembled a jellyfish visually but did not emit any other cues, such as chemical or auditory cues, this incident supports the use of visual cues in mid-water foraging by loggerhead turtles. Anthropogenic debris is commonly found in the digestive tracts of loggerhead turtles from various habitats (e.g., [80], [81]), including our study site [82]. However, in this case the turtle did not bite the plastic bag, although it approached as closely as it could touching the bag before making a final judgement. It may be that the turtle used other cues, such as chemical, tactile, and/or visual cues (e.g., the lack of pulsation) to discriminate the bag from gelatinous prey.

### Conclusions

By simultaneously collecting video and high-resolution 3D data, we examined mid-water foraging behaviour in loggerhead turtles, revealing that it is characterized by a distinct deceleration associated with the capture and handling of sluggish gelatinous prey. Based on this characteristic, our study indicates the possibility of using deceleration in swim speed as a proxy for mid-water foraging events. In addition, by examining 3D movements in detail, our study provides new insights into how loggerhead turtles forage, in the context of the sensory ecology involved in prey finding.

## Supporting Information

Figure S1 **Histogram of foraging depth showed unimodal pattern with peak at near 20 m.**
(TIF)

Table S1 **GLMM models with factors affecting foraging depth, presence of turning point and approach distance.**
(DOCX)

Movie S1 **A loggerhead turtle foraged on a sea nettle,** ***Chrysaora melanaster*** **.**
(MP4)

Movie S2 **A loggerhead turtle foraged on a chain of siphonophores.**
(MP4)

Movie S3 **An encounter with a plastic bag by a loggerhead turtle.**
(MP4)
