# Supplementary figures and images for: Loggerhead Turtles (Caretta caretta) Use Vision to Forage on Gelatinous Prey in Mid-Water
Source: PLoS One. 2013 Jun 12;8(6):e66043. doi: 10.1371/journal.pone.0066043 (PMC3680403; doi:10.1371/journal.pone.0066043)

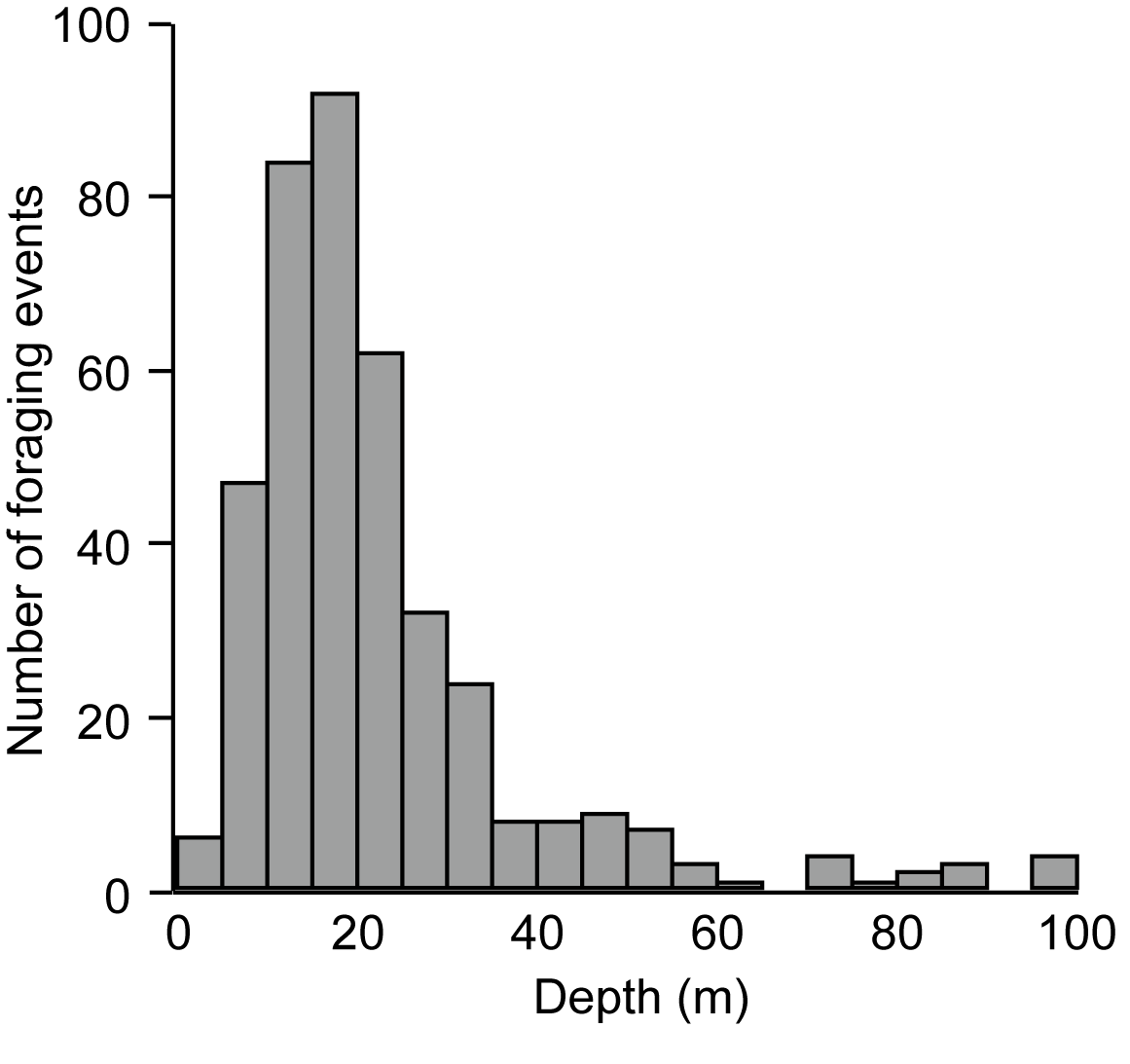

Supplement: Figure S1 — Histogram of foraging depth showed unimodal pattern with peak at near 20 m. (TIF) [file pone.0066043.s001.tif]
