# Supplementary material for: Loggerhead Turtles (Caretta caretta) Use Vision to Forage on Gelatinous Prey in Mid-Water
Source: PLoS One. 2013 Jun 12;8(6):e66043. doi: 10.1371/journal.pone.0066043 (PMC3680403; doi:10.1371/journal.pone.0066043)
Supplement: Table S1 — GLMM models with factors affecting foraging depth, presence of turning point and approach distance. (DOCX) [file pone.0066043.s002.docx]

**Table S1** GLMM models with factors affecting foraging depth, presence of turning point and approach distance.

| GLMM model |  | AIC | ΔAIC |
| --- | --- | --- | --- |
| (1) Foraging depth | ~ 1 | 163.5 | 0.0 |
|  | ~ Day/Night (DN) | 165.4 | 1.9 |
|  | ~ True/Presumptive (TP) | 165.5 | 2.0 |
|  | ~ DN + TP | 167.4 | 3.9 |
| (2) Presence of turning point | ~ 1 | 517.5 | 3.1 |
|  | ~ Foraging depth (dep) | 519.3 | 4.9 |
|  | ~ DN | 514.4 | 0.0 |
|  | ~ TP | 516.9 | 2.5 |
|  | ~ dep + DN | 516.2 | 1.8 |
|  | ~ dep + TP | 518.7 | 4.3 |
|  | ~ DN + TP | 515.8 | 1.4 |
|  | ~ dep + DN + TP | 517.7 | 3.3 |
| (3) Approach distance | ~ 1 | 257.0 | 3.6 |
|  | ~ dep | 253.8 | 0.4 |
|  | ~ DN | 256.5 | 3.1 |
|  | ~ TP | 257.3 | 3.9 |
|  | ~ dep + DN | 253.4 | 0.0 |
|  | ~ dep + TP | 253.7 | 0.3 |
|  | ~ DN + TP | 258.0 | 4.6 |
|  | ~ dep + DN + TP | 254.6 | 1.2 |
